# The Effect of Heavy Ion Irradiation on the Forward Dissolution Rate of Borosilicate Glasses Studied In Situ and Real Time by Fluid-Cell Raman Spectroscopy

**DOI:** 10.3390/ma12091480

**Published:** 2019-05-07

**Authors:** Mara Iris Lönartz, Lars Dohmen, Christoph Lenting, Christina Trautmann, Maik Lang, Thorsten Geisler

**Affiliations:** 1 Institut für Geowissenschaften und Meteorologie, Universität Bonn, Poppelsdorfer Schloss, Meckenheimer Allee 169, 53115 Bonn, Germany; cl@uni-bonn.de (C.L.); tgeisler@uni-bonn.de (T.G.); 2 SCHOTT AG, Hattenbergstr. 10, 55122 Mainz, Germany; lars.dohmen@schott.com; 3 GSI Helmholtzzentrum, 64291 Darmstadt and Technische Universität Darmstadt, 64287 Darmstadt, Germany; c.trautmann@gsi.de; 4 Department of Nuclear Engineering, University of Tennessee, Knoxville, TN 37996, USA; mlang2@utk.edu

**Keywords:** borosilicate glass corrosion, heavy ion irradiation, in situ fluid-cell Raman spectroscopy, forward dissolution rate

## Abstract

Borosilicate glasses are the favored material for immobilization of high-level nuclear waste (HLW) from the reprocessing of spent fuel used in nuclear power plants. To assess the long-term stability of nuclear waste glasses, it is crucial to understand how self-irradiation affects the structural state of the glass and influences its dissolution behavior. In this study, we focus on the effect of heavy ion irradiation on the forward dissolution rate of a non-radioactive ternary borosilicate glass. To create extended radiation defects, the glass was subjected to heavy ion irradiation using ^197^Au ions that penetrated ~50 µm deep into the glass. The structural damage was characterized by Raman spectroscopy, revealing a significant depolymerization of the silicate and borate network in the irradiated glass and a reduction of the average boron coordination number. Real time, in situ fluid-cell Raman spectroscopic corrosion experiments were performed with the irradiated glass in a silica-undersaturated, 0.5 M NaHCO_3_ solution at temperatures between 80 and 85 °C (initial pH = 7.1). The time- and space-resolved in situ Raman data revealed a 3.7 ± 0.5 times increased forward dissolution rate for the irradiated glass compared to the non-irradiated glass, demonstrating a significant impact of irradiation-induced structural damage on the dissolution kinetics.

## 1. Introduction

Borosilicate glass is the favored material for the geological disposal of high-level nuclear waste (HLW) from the reprocessing of spent fuel used in nuclear power plants [1,2,3]. Vitrification of HLW is currently the treatment of choice for immobilization of radionuclides for the following reasons: (a) high capability of glass to reliably incorporate a wide spectrum of isotopes with different ionic charges and sizes, (b) simple and economic production technology adapted from glass manufactures, (c) small volume of the resulting waste form, (d) high chemical durability of waste form glasses in contact with natural waters, and (e) high tolerance of these glasses to self-irradiation damage. Vitrification is also attracting great interest for other types of wastes, such as operational radioactive wastes from nuclear power plants as well as radioactive and toxic legacy waste from medicine [4]. In a deep geological disposal, the vitrified nuclear waste can come into contact with infiltrating ground waters once the protective metallic containers are broken or corroded. Numerous laboratory experiments were conducted to identify the key mechanisms that lead to glass breakdown in aqueous solutions and to determine the reaction kinetics [1,2,5,6,7,8,9,10,11,12]. There is still an intensive debate on the reaction and transport mechanisms controlling glass alteration and the formation of silica-based surface alteration layers (SALs) over geological time scales, reflected by the formulation of several different mechanisms, including the fundamentally different and highly debated leaching and interface-coupled dissolution-precipitation (ICDP) models [5,6,7,8,9,10,11,12,13]. While the leaching model is based on the fact that the SAL forms by diffusion-controlled chemical leaching of the glass, the ICDP model is based on the notion that the glass initially dissolves congruently until a surface solution boundary layer is supersaturated with amorphous silica, which eventually precipitates at the surface of the glass. Both, glass dissolution and silica precipitation are coupled in space and time, resulting in the formation of an inwardly migrating dissolution-precipitation front. In a recent study, however, Lenting et al. [14] presented a unifying model based on an ICPD mechanism as the SAL forming process, but also includes an interdiffusion zone that develops ahead of the ICDP front inside the glass once the ICDP reaction slowed down due to chemical transport limitations.

**Figure 1:**
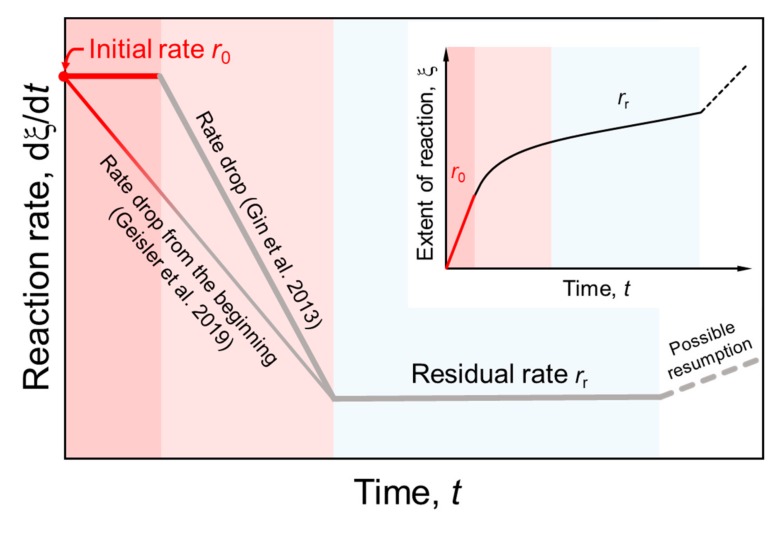
Kinetic regimes of glass corrosion that have been identified to date [2], illustrated by a plot of the reaction rate and the extent of reaction, ξ (inset diagram), as a function of time. Here, ξ represents the amount of glass dissolved into solution or transformed into a silica-based surface layer (SAL). In the present work, we are mainly concerned with the initial reaction rate, *r*_0_, that represents the forward dissolution rate of the glass. Gin and coworkers [2] proposed a constant *r*_0_ for a certain time until an SAL is formed. In contrast, Geisler et al. [21] recently observed a linearly decreasing *r*_0_ over time already from the beginning of the reaction without the formation of an SAL.

Regardless of the mechanistic details, several kinetic stages have been identified during nuclear glass corrosion as schematically shown in Figure 1 [2,15,16]. During the first kinetic stage, the corrosion rate *r*_0_, defined as the amount of glass lost per unit time, is assumed to be constant until it linearly drops to a residual rate *r*_r_ [3,16,17]. However, Ojovan et al. [4] proposed an exponential decrease of the elemental release rate already from the beginning of reaction. A late-stage resumption of the corrosion rate can occur under yet unclear circumstances, which are in focus of current research [18,19,20].

Using a unique experimental setup that allows the in situ observation of the corrosion process by fluid-cell confocal Raman spectroscopy, Geisler et al. [21] found a linear decrease of the dissolution rate with time already from the beginning of the reaction (Figure 1). The in situ experiments with a ternary Na borosilicate glass revealed an initially continuously retreating glass-water interface without any evidence for the formation of an SAL, suggesting that the glass initially dissolved congruently and that affinity effects control the initial rate decrease until a several micrometer-thick SAL had formed and the rate dramatically dropped to zero. The initial dissolution rate *r*_0_ was determined from the intercept of a linear fit with the rate axis and interpreted to be the forward dissolution rate of the investigated borosilicate glass far away from equilibrium under the given physicochemical conditions (Figure 1).

To reliably assess the chemical durability of nuclear waste glasses over geological time scales, it is critical to understand the impact of self-irradiation damage on the glass properties and particularly its effect on the aqueous dissolution kinetics. Self-irradiation damage from alpha-decay falls into two categories: (i) the transfer of the energy from the damaging energetic alpha particle to the electrons of the glass (ionization and electronic excitations); and (ii) the transfer of energy to the atomic nuclei, primarily by ballistic processes involving elastic collisions. The more massive, but low energy (70–100 keV) α-recoil particles account for most of the total number of displacements produced by ballistic processes in HLW glasses. The α-recoils lose nearly their entire energy in elastic collisions over a very short range, producing highly localized damage (displacement cascades) [22,23,24,25]. For the simulation of the absorbed dose from both types of electronic or nuclear collisions, the irradiation of non-radioactive glass with heavy or light ions is a scientifically valuable method [13]. In particular, ion accelerator experiments allow an assessment of the effect of a single process on the material structure that is produced during the radioactive decay under well-controlled conditions. The ability to isolate defect mechanisms is crucial to the study of complex irradiation-damage problems, where both competing and synergistic contributions from the irradiation environment of temperature and stress exist [26].

**Figure 2:**
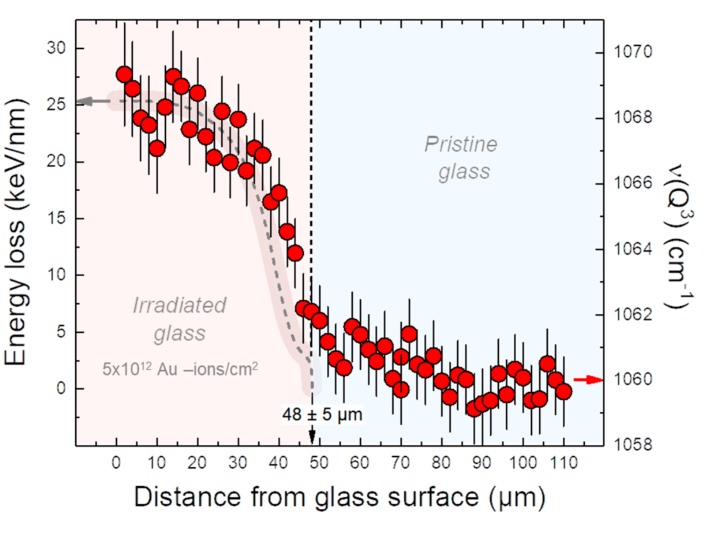
The energy loss of 4.8 MeV/u Au ions calculated with the SRIM code [27] (dashed gray line) and the frequency of the Q^3^ Si-O stretching mode (red circles) as a function of depth. Note the good agreement between the SRIM calculation, indicating a maximum Au penetration depth of 48 ± 5 µm, and structural changes reflected by the shift of the frequency of the Q^3^ band.

In this study, Au ions of ~1 GeV energy were chosen to evaluate the effect of purely electronic collisions on the glass network and ultimately on the initial forward dissolution rate *r*_0_. The use of swift heavy ions has the advantage that a relatively thick layer of irradiated material can be produced (Figure 2). For this, we performed two in situ and real time, fluid-cell Raman spectroscopic corrosion experiments with an ion-irradiated Na borosilicate glass in a 0.5 mol NaHCO_3_ solution, following the method described in detail in Geisler et al. [21]. These authors already carried out corrosion experiments with non-irradiated glass samples of the same composition and under very similar physicochemical conditions, which serve as reference. In addition, we repeated one experiment with a non-irradiated sample to test the reproducibility of the method.

## 2. Materials and Methods

### 2.1. Glass Synthesis

The irradiation experiment was carried out with a polished, homogenous, ternary Na borosilicate glass (TBG) coupon (13 × 5 × 3 mm^3^) that was prepared from a second production batch of the glass used by Geisler et al. [18]. The nominal composition of the glass was 57.9 wt.% SiO_2_, 19.7 wt.% Na_2_O, and 22.3 wt.% B_2_O_3_ [21]. It was synthesized from SiO_2_, B_2_O_3_, and Na_2_CO_3_ precursors in a platinum crucible at 1400 °C. The glass was molten a second time after crushing and milling it in a ball mill to ensure chemical homogeneity. After quenching the melt in a pre-heated stainless steel mold, it was tempered at 560 °C for 6 h and then letting it cool down over night by switching off the oven.

### 2.2. Heavy Ion Bombardment

One side of the polished sample was irradiated at room temperature and at normal incidence with ^197^Au ions at the beamline M3 of the UNILAC at the GSI Helmholtz Center for Heavy Ion Research in Darmstadt, Germany. The generated ^197^Au ions had an energy of 4.8 MeV/u (with u being the mass unit) and an effective energy of 4.5 MeV/u at the sample surface, i.e., a kinetic energy of 945.6 MeV. The samples were irradiated to a fluence of 5 × 10^12^ ions/cm^2^ with an uncertainty in the order of 10–20%. The penetration depth of the ^197^Au ions in the Na borosilicate glass (2.51 g/cm^3^) was 48 ± 5 µm as calculated with the SRIM 2013.00 code [27] (Figure 2). For the two corrosion experiments, the irradiated glass coupon was cut in two smaller coupons with a size of about 6 × 5 × 3 mm^3^ and 7 × 5 × 3 mm³, respectively.

### 2.3. Raman Spectroscopy

All Raman measurements were conducted with a high resolution Horiba Scientific HR800 confocal Raman system at the Institute for Geosciences and Meteorology of the University in Bonn, Germany. A 2 W solid state Nd:YAG laser (532.09 nm) with about 600 mW power at the sample surface was used. The scattered light was detected with an electron multiplier charge-coupled device detector after having passed a 1000 µm confocal aperture and a 100 µm spectrometer entrance slit, and being dispersed by a grating of 600 grooves/mm. A 100× long working distance (LWD) microscope objective with a numerical aperture of 0.8 was used for all measurements, yielding an empirically determined lateral resolution of about 6 µm at the depth of the in situ Raman measurements [21]. The Raman signal was measured in the wavenumber ranges from 200 to 1800 and from 2800 to 4000 cm^−1^. The first frequency range includes a Ne line at 1706.06 cm^−1^ that was recorded as an internal wavenumber standard to correct each spectrum for any spectrometer shift during long term measurements of up to 75 h (resulting from ±0.5 °C temperature variations in the laboratory) by placing a Ne lamp in the beam path of the scattered light.

To determine the relative fractions of different structural glass units in the non-irradiated and irradiated glass from Raman intensities, the measured Raman intensities *I*(ν) were corrected for (1) the wavelength-dependent instrumental sensitivity (white light correction); (2) the thermal population of the excited states by the Bose–Einstein temperature factor *B* = [1 − exp(−*h*ν*c*/*kT*)] with *h*, *k*, *c*, and *T* being the Planck and Boltzmann constant, the speed of light, and the absolute temperature, respectively; (3) the scattering factor *q*_s_ = (ν_e_ – ν)^–3^ with ν_e_ and ν being the excitation wavenumber and Raman shift, respectively; (4) the frequency factor ν; and, finally, (5) for background signals (stray light, fluorescence) that were fitted with a third order polynomial function. The corrected spectrum, the so-called reduced or *R*(ν) spectrum, is directly proportional to the relative scattering activity in terms of mass-weighted normal coordinates and thus most closely matches the vibrational density of states.

### 2.4. Experimental Set-Up and Determination of Glass Retreat/Dissolution Rate

**Figure 3:**
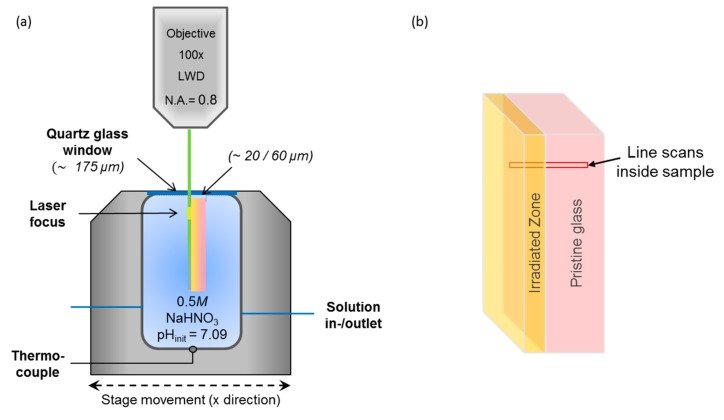
(**a**) Schematic drawing of the fluid cell (not to scale) used in this study, and (**b**) the location of the Raman spectroscopic line scans cross the heavy ion bombarded (5 × 10^12^ Au ions/cm^2^) surface layer into the non-irradiated glass.

Three in situ experiments were performed with two irradiated and one non-irradiated reference glass coupon under similar physiochemical conditions using a home-made fluid-cell (Figure 3a) [21]. Before each experiment, the fluid-cell was disassembled, cleaned with 35% HCl solution and rinsed with MilliQ^©^ water. The glass coupon was placed in a sample holder that is integrated in the sealing cap. In the closed fluid-cell the accurate positioning of the glass coupon, perpendicular to the fused silica window, is of great importance for the reliable determination of the glass retreat, i.e., the glass dissolution rate. Furthermore, the distance between the window and the glass sample surface was kept as small as possible in order to minimize corrosive processes in this region and to obtain the best possible spatial resolution. In the first experimental set up the distance between the fused silica window and the sample surface was ~60 µm. In the second experiment the distance was ~20 µm. The fluid-cell was then filled with 13.0 ± 0.2 mL of 0.5 M NaHCO_3_ solution (initial pH of 7.1 at 85 °C; [21]) and heated to nominal temperature of 90 °C. Due to a slight temperature gradient in the cell, the actual temperature at the position of the Raman measurements was between about 80 and 85 °C (Table 1), which was accurately determined from the temperature-related frequency shifts of the ν_5_(HCO_3_) band as described in detail in [21].

Point-by-point parallel Raman line scans were performed every ~2 h about 100 µm below the surface of the glass monolith with a 2-µm step size by automatically moving the stage in y–x direction (Figure 3a). The total counting time for each point was about 25 s, involving the two spectral windows that were each measured for 2 × 6 s. For the determination of the glass retreat as a function of time, the integrated total intensity of the Q^n^ species between 1000 and 1250 cm^−1^ was determined from white light- and background-corrected *I*(ν) spectra. The resulting intensity profiles across the glass-water interface were than fitted with an exponential function from which the glass retreat was determined with an error better than about ± 1 µm. For a more detailed description of the determination of the glass retreat we refer to Geisler et al. [21].

## 3. Results

### 3.1. Structural State of the Irradiated Glass Samples

**Figure 4:**
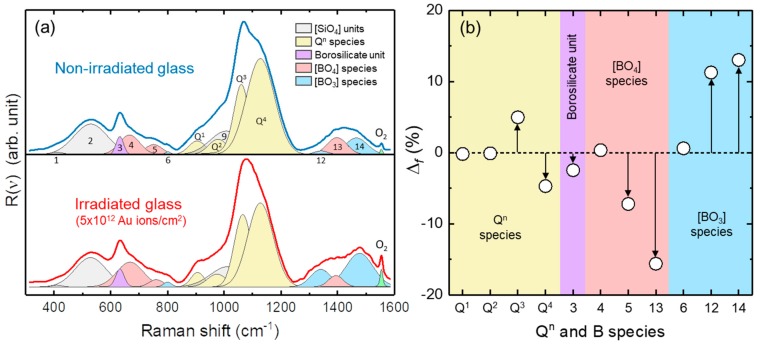
(**a**) Representative background-corrected *R*(ν) Raman spectra (average of 10 individual spectra) from the non-irradiated glass (upper spectrum) and the irradiated surface layer (lower spectrum) of the ternary Na borosilicate glass (TBG) used in this study for dissolution experiments along with the result of a least-squares fit with 15 Gauss–Lorentz functions. The assignment of the Raman bands to a vibrational mode of a structural glass unit and to O_2_ is indicated by colored Gauss-Lorentz profiles. The different glass bands are labeled by numbers and by the Q^n^ notation in the cases of the stretching motions of [SiO_4_] groups with n = 1, 2, 3, or 4 bridging oxygen atoms. Note the significantly higher intensity of the O_2_ band near 1555 cm^−1^ in the spectrum from the irradiated sample. (**b**) Diagram showing the difference, Δ*_f_*= *f*_irr_ − *f*_non-irr_, between the intensity fraction of different silicate- and borate-related bands of the irradiated, *f*_irr_, and non-irradiated, *f*_non-irr_, TBG. The fractions were determined from the integrated intensities and the intensity sum of the respective Raman bands that are shown and labeled in Figure 4a.

The Au irradiation of the pristine glass with a kinetic energy of ~950 MeV (at the surface) and a fluence of 5 × 10^12^ Au ions/cm^2^ did not result in any macroscopically visible changes of the sample. However, an effect of heavy ion irradiation on the structure of the ternary Na borosilicate glass (TBG) was detected by Raman spectroscopy. Figure 4a shows a representative, average *R*(ν) Raman spectrum from both the irradiated and non-irradiated TBG, which were extracted from the first Raman point-by-point line scan from the irradiated glass surface into the non-irradiated glass that was measured at the beginning of each experiment (Figure 3b). To quantify spectral changes after irradiation, which are already evident by visible inspection of the Raman spectra, we fitted the background-corrected *R*(ν) spectra shown in Figure 4a with 15 Gauss-Lorentz functions in the wavenumber range between 300 and 1600 cm^−1^. It is noted that the least-squares fitting procedure of silicate glass vibrational bands is still highly debated [28]. For instance, some researchers favor the use of Gauss functions to fit Raman bands of silicate glasses (e.g., [29,30]), whereas others use a convolution of a Gauss and Lorentz function (e.g., [28,31]), which takes into account phonon dumping on the density of states which is particularly relevant in disordered materials where phonon life times are short [28]. Furthermore, the broad vibrational bands from silicate glasses make it often difficult to decide how many vibrational modes contribute to a broad band profile. In this study, we applied the deconvolution procedure proposed by Manara et al. [31], who used 14 Gauss-Lorentz functions to fit the Raman spectra of a series of ternary Na borosilicate glasses in the wavenumber range between 300 and 1600 cm^−1^. The 15th band observed in this study was located near 1555 cm^−1^ and reflects the stretching mode of molecular oxygen. The assignment of the 14 vibrational modes of typical structural units in Na borosilicate glasses, which is given in Figure 4a, was also adapted from Manara et al. [31], but their assignment also bases on earlier work (e.g., [32,33,34]).

The band near 495 cm^−1^ in the spectrum of the non-irradiated glass—also called R band (band 2 in Figure 4a)—was assigned to bending and rocking modes of Si–O–Si bonds. A wavenumber upshift by 10 cm^−1^ was observed for this band in the spectra from the irradiated part of the sample, which is in perfect agreement with findings of Mir et al. [35] on borosilicate glass irradiated with ^129^Xe ions, and indicates a decreased mean Si-O-Si angle due to irradiation (Figure 4a). Vibrational stretching motions of the silicate network are represented by bands near 905, 978, 1061, and 1128 cm^−1^ in the non-irradiated TBG, which have been assigned to Q^1^, Q^2^, Q^3^, and Q^4^ species in the silicate network, respectively (the superscript refers to the number of bridging oxygen atoms). Another Raman band contributes to the overall profile in this wavenumber region, namely a band near 1008 cm^−1^ (band 9 in Figure 4a) that was considered to represent Si–O^0^ bridging oxygen stretching motions [34] or alternatively vibrations of structural units associated with Na^+^ ([32,33]). In general, the structural units with a high degree of polymerization contribute to the high-frequency side of the Q^n^ region, whereas the units with a low degree of polymerization contribute to the low frequency side [35]. Here, we observed a 5.0 ± 0.5% increase of the Q^3^ fraction in the irradiated part of the TBG (Figure 4b), and a simultaneous −4.7 ± 0.5% decrease of the Q^4^ fraction, clearly indicating a depolymerization of the SiO_4_ glass network. On the other hand, the Q^1^ and Q^2^ fractions did not significantly change. The Q^3^ band showed the largest frequency shift (~8 cm^−1^; Figure 2) among the Q^n^ bands in response to Au ion irradiation. The variation of the frequency of the Q^3^ band as a function of depth perfectly mimics the energy loss curve for Au ions in the TBG calculated by the SRIM code (Figure 2).

We consider the Raman bands assigned to the different borate species in borosilicate glasses. The pronounced band near 632 cm^−1^ (band 3 in Figure 4a) has been associated with bending motions of danburite-like borosilicate ring units [31]. The intensity fraction of this band on the total intensity of all Raman bands assigned to boron species (Figure 4a) was 2.5 ± 0.4% lower in the spectra from the irradiated surface layer (Figure 4b). However, the intensity fraction of the bands near 1342 and 1466 cm^−1^ (bands 12 and 14 in Figure 4a, respectively), that were assigned to the BO_3_ units in boroxol rings, was significantly higher in the spectra from the irradiated surface layer (Figure 4b). This change was complemented by significantly lower intensity fractions of BO_4_-related bands (Figure 4b), i.e., bands 4, 5, and 14 (Figure 4a). A detailed inspection of the spectra furthermore revealed that the broad band profile between 1250 and 1650 cm^−1^ in the spectra from the irradiated surface layer were characterized by a number of new inflection points. This suggests that apart from an average reduction of the boron coordination number, the B–O bond length ordering has also changed due to irradiation. These changes are clear evidence for significant structural alterations of the borate sub-network as a result of heavy ion irradiation. In this respect, we also noted a significantly higher intensity of the O_2_ band in the spectrum from the irradiated compared to the non-irradiated part of the samples (Figure 4a). Whereas the O_2_ band in the spectra from the non-irradiated glass areas solely stems from air located between the glass surface and the objective, the higher intensity of the O_2_ band in the spectra from the irradiated part of the TBG indicates the occurrence of additional molecular oxygen within the irradiated glass structure. This is consistent with Raman spectroscopic results of Mir et al. [32], who also observed the O_2_ band after irradiation of a ternary Na borosilicate glass with ^129^Xe ions.

### 3.2. Glass Retreat and forward Dissolution Rate

**Figure 5:**
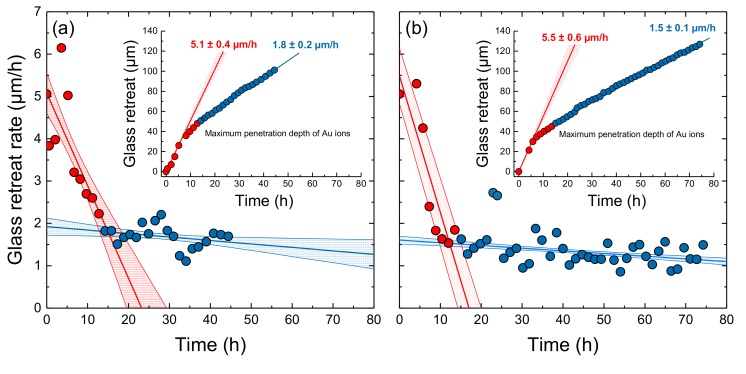
The dissolution rate and glass retreat (inset) as a function of time for (**a**) the first and (**b**) the second experiment with the irradiated TBG. The rate data clearly define two distinct trends that were individually fitted with a linear function. Light blue data points in Figure 5b were identified as outliers and excluded from the fit. The two trends can be related to the congruent (stoichiometric) dissolution of the irradiated surface layer (red symbols) and the underlying non-irradiated glass (blue symbols). The intercept of the red line with the y axis defines the forward dissolution rate, *r*_0_, of the irradiated glass, whereas the intercept of the blue line with the red line gives the forward dissolution rate of the non-irradiated glass and defines the time when glass dissolution reached the non-irradiated part of the glass.

**Figure 6:**
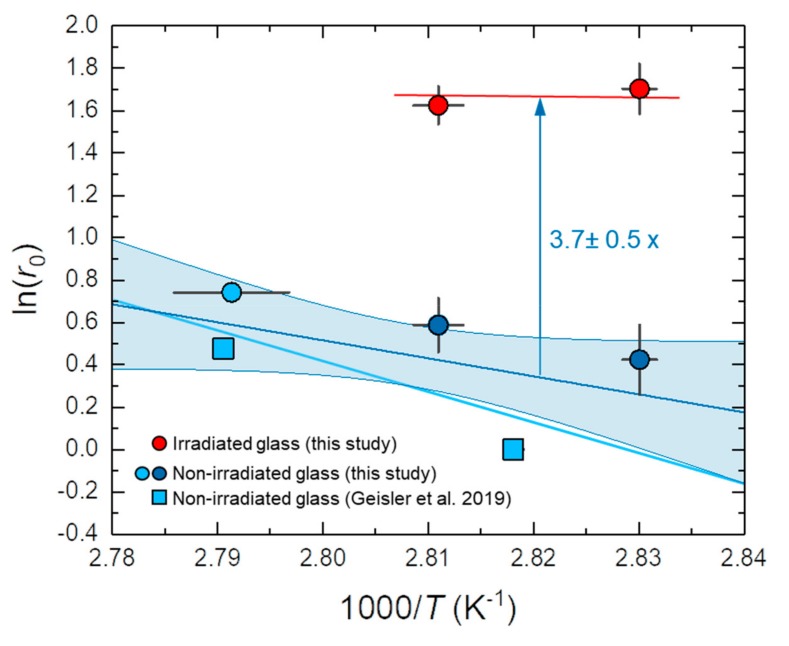
Arrhenius diagram with the initial or forward dissolution rates *r*_0_ obtained for the non-irradiated (blue symbols) and irradiated (red symbols) TBG. Data from Geisler et al. [21] are also plotted. The dark and light blue lines represent an unweighted and an error-weighted linear fit to the data, respectively. For the unweighted fit the 1-sigma confidence interval is also shown as we use this fit to estimate *r*_0_^irr^/*r*_0_ and its error.

Two in situ fluid-cell Raman spectroscopic corrosion experiments were conducted with the irradiated TBG samples for ~45 and ~75 h (Figure 5a,b). Another short-term experiment was performed with a non-irradiated TBG coupon for 7 hours to test the reproducibility of the method by comparing results with those from Geisler et al. [21]. The results are summarized in Table 1. Both experiments with irradiated samples started with a remarkably higher initial forward dissolution rate than experiments with the non-irradiated glass (Figure 5), clearly demonstrating a significant effect of radiation damage and associated structural modifications on the glass dissolution kinetics. In agreement with results from Geisler et al. [21], an approximately linear decrease of the glass retreat rate is observed from the beginning of both experiments until about 12 to 15 h, when a sharp rate drop occurred. Two rate-time regimes can thus be distinguished that were each individually fitted with a linear function (red and blue lines in Figure 5). Since the drop of the glass retreat rates occurred just about when the dissolution of the glass reached the maximum penetration depth of the Au ions (Figure 5 insets), the drop can reliably be linked to the time when the irradiated surface layer is dissolved completely so that the non-irradiated glass is exposed to the solution. In this respect, it is also noteworthy that first silica signals were observed only in the second, longer experiment after about 60 h, which is fully in line with findings of Geisler et al. [21]. Thus, extrapolation of the linear fits to the initial rate data of both experiments (red lines in Figure 5) to *t* = 0 delivered the initial forward dissolution rate of the irradiated glass, i.e., *r_0_^irr^* = 5.1 ± 0.4 and 5.5 ± 0.6 µm/h, respectively (Figure 5; Table 1). The relative large uncertainty of the dissolution rates at the beginning reflects the fast dissolution kinetics with respect to the scanning time and thickness of the irradiated surface layer. The intercept of the best fit line of both kinetic regimes should correspond to the initial dissolution rate of the non-irradiated TBG. It also gives the time when the rate drop occurred, i.e., after 15 ± 3 and 12 ± 2 h, respectively (Figure 5). Indeed, we obtain *r_0_* values of 1.8 ± 0.2 and 1.5 ± 0.1 µm/h that are comparable with the forward dissolution rates obtained from the non-irradiated TBG (Table 1). Figure 6 shows a compilation of all data in an Arrhenius diagram to consider the slightly different temperatures of the experiments (Table 1).

The significantly higher forward dissolution rate of the irradiated glass compared to the non-irradiated counterpart is immediately apparent, but the rate data from the non-irradiated TBG themselves are highly scattered. This excess scatter may reflect (i) the slightly different chemistry of the glass coupons used in this study compared with those used by Geisler et al. [21], and/or (ii) the observation that the dissolution rate may locally vary as reflected by etch pits often observed in glass dissolution studies [12]. An unweighted linear least squares fit to the data yielded a slope that corresponds to an activation energy of 71 ± 66 kJ/mol, whereas the slope of an error-weighted linear fit yielded an activation energy of 116 ± 83 kJ/mol (Figure 6). Both values are statistically not significantly different from zero, but are nevertheless in the typical range of activation energies for hydrolysis reactions at borosilicate glass surfaces that were calculated from first principles density functional theory (DFT) simulations and measured in neutral solutions ([36], and references therein). However, it is not expected that the dissolution rates obtained for the non-irradiated glass from the experiment with the surface-irradiated TBG perfectly fit to those obtained with non-irradiated glass coupons, since at the time when the irradiated surface layer is completely dissolved, the solution chemistry is different from the initial chemistry. At that time the solution was no longer free of silica (but still undersaturated with respect to amorphous silica) and the pH near the surface changed, so the chemical affinity or driving force for the dissolution reactions also changed [34]. In any case, to obtain a reliable activation energy for the dissolution of this TBG under close to neutral conditions, more experiments under a wider temperature range are necessary. The unweighted fit yielded a ratio between *r*_0_^irr^ and *r_0_* of 3.7 ± 0.5 (Figure 6). Thus, an almost four-fold increase of the forward dissolution rate was observed for the irradiated TBG.

## 4. Discussion and Conclusions

In this study, the irradiated glass structure was found to dissolve 3.7 ± 0.5 times faster than the corresponding non-irradiated glass, verifying previous studies that also reported an increased dissolution kinetics of radiation-damaged silicate glasses [37,38,39]. In contrast, other studies claim that neither the alpha activity nor the alpha decay dose has a significant impact on the initial dissolution rate [40,41]. The reasons for such a disagreement are not evident and should be subject of future investigations, particularly given the importance of this knowledge to assess the chemical durability of nuclear glasses in a natural nuclear repository. Raman spectroscopic measurements of the Au-irradiated TBG have revealed (1) a significant modification of the short-range order around the main network formers, (2) a decrease of the average boron coordination number, (3) an accumulation of molecular oxygen, and (4) an increase of the Q^3^ fraction at the expense of the Q^4^ species, indicating depolymerization of the silicate network due to radiation damage. Such structural and chemical modifications are in line with findings of Mir et al. [35] and known to be accompanied by changes in the physical properties, including hardness and Young’s modulus, density, and fracture toughness [23,37,42,43,44,45,46,47]. Our study reveals that such structural modifications due to irradiation have a profound effect on the glass dissolution property.

The increased dissolution kinetics can principally be explained by the less interlinked, irradiated glass network that offers a larger free volume for the hydrolysis of Si–O and B–O bonds in the glass by hydrogen species from solution. During heavy ion bombardment, several micrometer-long cylindrical damage trails, so-called ion tracks [48], are created, which have been made visible by transmission electron microscopy [24] and atomic force microscopy [49]. In particular, the Si–O and B–O bonds inside the ion tracks are likely high energy sites that can preferentially be hydrolyzed. In our samples, these ion tracks must be parallel oriented and elongated in the direction of the dissolution front movement due to the direction of bombardment. Considering the long-range ballistic character of heavy ions in the material, it would be interesting to test whether the dissolution kinetics depends on track orientation? This question directly relates to the general debate about the scientific value of external heavy ion irradiation experiments for the simulation of α-recoil damage [37]. Charpentier et al. [50], for example, observed a higher damage level in silicate glasses irradiated with Au ions than in short-lived actinide-doped glasses that accumulated an equivalent dose of recoil events. A possible explanation for this observation could be partial self-healing processes which arise from alpha particles involved in alpha decay events [49]. External irradiation with energetic heavy ions may also induce the removal of alkali ions even from depths that correspond to the ion penetration depth [51]. At high fluences, the entire near surface region may be depleted of alkali ions to depths even exceeding the ion range [51]. However, for our samples electron microprobe measurements across the irradiated surface layer did not reveal any Na loss from areas deeper than ~1 µm (lateral resolution of the measurements) below the surface. Moreover, a very critical point is that in real case scenarios, radiolysis reactions in the surface boundary solution may significantly affect the glass dissolution kinetics as also observed to affect the dissolution of UO_2_, respectively spent fuel [39,52,53].

All these observations challenge the assignability of results from heavy ion irradiated samples to the real world of self-irradiation in nuclear glasses. However, the irradiation with swift heavy ions requires less experimental effort and represents an effective approach to simulate radiation damage over micrometer length scale and thus allows studying the effects of radiation damage on the long-term durability of borosilicate glasses. On the other hand, experiments with borosilicate glasses doped with short- and long-lived actinide and/or fission products appear to be most important for the assessment of the long-term durability of a nuclear glass in a natural repository and, not to forget, to compare the results with those obtained from externally irradiated samples. Here, fluid-cell Raman spectroscopy can potentially open up new possibilities to study the corrosion of nuclear glass in situ without disturbing or interrupting the reaction. As shown by Geisler et al. [21], and partly in this work, fluid-cell Raman spectroscopy thereby provides a wealth of chemical and structural information with a spatial resolution at the micrometer-scale. Moreover, light stable isotopes, such as ^2^H and ^18^O, can be used to in situ trace distinct reaction and transport processes, as demonstrated by Geisler et al. [21], who quantified the transport of molecular water through a silica-based surface alteration layer by using a ^2^H-labelled solution. A single experiment thus delivers the information of numerous quench experiments with different samples so that complicated sample syntheses and preparation that require a radiation-protected environment, as well as costs and new nuclear waste, are minimized. Such experiments can potentially also be performed over longer durations to be able to also investigate the corrosion process of nuclear waste glasses as they are kinetically more stable against aqueous corrosion than the glass used in the present study.

## Figures and Tables

**Table 1:** Summary of forward dissolution rates of non-irradiated, *r*_0_, and irradiated TBG, *r*_0_^irr^, obtained by in situ fluid-cell Raman spectroscopy. The uncertainties were obtained from the least-squares fit (Figure 5) and are given at the 1-sigma level.

Sample	Non-Irradiated TBG			Irradiated TBG	
T (°C) *r*_0_^irr^ (µm/h) *r*_0_ (µm/h)	85.2 ± 0.2 - 1.61 ± 0.04	81.7± 0.1 - 1.00 ± 0.03	85.1 ± 0.7 - 2.10 ± 0.03	80.0 ± 0.2 5.1 ± 0.4 1.8 ± 0.2	82.6 ± 0.3 5.5 ± 0.6 1.5 ± 0.1
Reference	[21]		This study		
